# 

**Published:** 1882-11

**Authors:** 


### *The American Academy of Dental Science.*

The fifteenth annual meeting of the American Academy of Dental Science was held at Young's Hotel, Boston, on October 25th. The morning session was chiefly devoted to the regular business and to the election of the following-named officers: President, Dr. G. T. Moffatt; vice president, Dr. J. H. Batcheller; recording secretary, Dr. H. F. Hamilton; corresponding secretary, Dr. E. B. Hitchcock; treasurer, Dr. E. H. Smith; librarian, Dr. H. C. Meriam; executive committee, Dr. C. P. Wilson, Dr. E. C. Briggs and Dr. J. S. Mason. Dr. S. J. Shaw, and Dr. F. E. Banfield were elected active fellows. The afternoon session opened with the annual address by Dr. Frank Abbot of

New York on "Dental Education." The paper was chiefly devoted to emphasizing the necessity of young men's hav- a thorough medical education as a preliminary to the study of dentistry, and the need of Massachusetts following the other States in obliging practising dentists to have a suitable degree or to pass an examination before a State examining board. Dr. W. H. Atkinson of New York read a paper on "Artificial Teeth," giving valuable ideas on the subject, and on the healing of tissues. He unqualifiedly condemned celluloid and vulcanite as base-plates. Dr. A. W. Buckland of Woonsocket, R. I., read a paper describing a method of filling by covering a lining of cement with amalgam while both are in a plastic state. He deprecates the use of gold and severe methods with patients needing gentle treatment. Resolutions were passed on the death of Dr. J. E. Fiske of Salem, and Dr. W. H. Allen of New York, both honorary fellows of the Academy. The annual dinner took place at five o'clock. Many distinguished dentists from other cities were present, and the meeting was one of the most valuable, both prefessionally and socially, ever held.

H. F. Hamilton,
*Recording Secretary.*

124 Commonwea.th Ave. Boston.

---

### EDITORIAL, ETC.

---

*Dental Education* —The winter or regular Sessions of the Dental Schools throughout the country have now fairly commenced,

### To Readers and Correspondents.

Communications intended for publication, and exchange journals and papers, should be addressed to the EDITOR OF THE AMERICAN JOURNAL OF DENTAL SCIENCE, 259 N. Eutaw St., Baltimore, Md. All letters relating to the business of the journal, or containing cash remittances, to be directed to Messrs. SNOWDEN & COWMAN. Articles for publication should be received by the editor at least three weeks previous to the first of the month on which the number in which it is designed for them to appear, is issued.

☞ The American Journal of Dental Science will contain fifty pages of reading matter in each number, making a volume of about

*Six Hundred* pages,

EXCLUSIVE OF ADVERTISEMENTS.

THE

### AMERICAN JOURNAL
OF
### DENTAL SCIENCE.

The *Journal* is issued on the first of each month and contains not less than *fifty* pages of reading matter in each number, exclusive of advertisements, making a volume of *six hundred* pages per annum.

In each number will be found Original Communications, Correspondence, Selected Articles, Monthly Summary, Bibliographical Notices and Editorial Matter, and every effort will be exerted to render the *Journal* worthy of the patronage of the Dental profession.

A generous co-operation is respectfully solicited from the members of the profession; and as the *Journal* has now a printing office of its own, which materially lessens the cost of its publication, it will be issued at the reduced subscription price of

**$2.50 Per Annum in Advance.**

Communications are respectfully solicited on all subjects pertaining to the practice of dentistry, as well as reports of cases occurring in dental practice.

### RATES OF ADVERTISING.

| | 1 MO. | 2 MO. | 3 MO. | 6 MO. | 12 MO. |
|---|---|---|---|---|---|
| 1 PAGE. | $15 00 | $22 50 | $30 00 | $50 00 | $100 00 |
| ½ " | 10 00 | 15 00 | 20 00 | 30 00 | 50 00 |
| ¼ " | 7 00 | 11 00 | 15 00 | 20 00 | 30 00 |
| ⅛ " | 5 00 | 8 00 | 11 00 | 15 00 | 20 00 |

All original communications designed for publication, works for review, new instruments for notice, exchanges, &c., should be directed to the editor, 259 N. Eutaw St., Baltimore, Md.

All advertisements and subscriptions should be addressed to

SNOWDEN & COWMAN,

*Publishers of the Am. Journal of Dental Science.*

86 W. FAYETTE ST. BET. CHARLES & LIBERTY STS.,

BALTIMORE, MD

